# Cutaneous stimulation of discrete regions of the sole during locomotion produces “sensory steering” of the foot

**DOI:** 10.1186/2052-1847-6-33

**Published:** 2014-08-08

**Authors:** E Paul Zehr, Tsuyoshi Nakajima, Trevor Barss, Taryn Klarner, Stefanie Miklosovic, Rinaldo A Mezzarane, Matthew Nurse, Tomoyoshi Komiyama

**Affiliations:** 1 Rehabilitation Neuroscience Laboratory, University Victoria, PO Box 3010 STN CSC, Victoria, BC V8W 3P1, Canada; 2 Department of Integrative Physiology, Kyorin University School of Medicine, 6-20-2 Shinkawa, Mitaka, Japan; 3 Laboratory of Signal Processing and Motor Control, College of Physical Education, University of Brasília, Brasília, Brazil; 4 Biomedical Engineering Laboratory, EPUSP, PTC, University of São Paulo, São Paulo, Brazil; 5 Nike Sport Research Laboratory, Beaverton, OR, USA; 6 Department of Health and Sports Sciences, Faculty of Education, Chiba University, Chiba, Japan; 7 Human Discovery Science, International Collaboration on Repair Discoveries (ICORD), Vancouver, BC, Canada; 8 Centre for Biomedical Research, University of Victoria, Victoria, BC, Canada; 9 Division of Medical Sciences, University of Victoria, Victoria, BC, Canada

## Abstract

**Background:**

While the neural and mechanical effects of whole nerve cutaneous stimulation on human locomotion have been previously studied, there is less information about effects evoked by activation of discrete skin regions on the sole of the foot. Electrical stimulation of discrete foot regions evokes position-modulated patterns of cutaneous reflexes in muscles acting at the ankle during standing but data during walking are lacking. Here, non-noxious electrical stimulation was delivered to five discrete locations on the sole of the foot (heel, and medial and lateral sites on the midfoot and forefoot) during treadmill walking. EMG activity from muscles acting at the hip, knee and ankle were recorded along with movement at these three joints. Additionally, 3 force sensing resistors measuring continuous force changes were placed at the heel, and the medial and lateral aspects of the right foot sole. All data were sorted based on stimulus occurrence in twelve step-cycle phases, before being averaged together within a phase for subsequent analysis.

**Methods:**

Non-noxious electrical stimulation was delivered to five discrete locations on the sole of the foot (heel, and medial and lateral sites on the midfoot and forefoot) during treadmill walking. EMG activity from muscles acting at the hip, knee and ankle were recorded along with movement at these three joints. Additionally, 3 force sensing resistors measuring continuous force changes were placed at the heel, and the medial and lateral aspects of the right foot sole. All data were sorted based on stimulus occurrence in twelve step-cycle phases, before being averaged together within a phase for subsequent analysis.

**Results:**

The results demonstrate statistically significant dynamic changes in reflex amplitudes, kinematics and foot sole pressures that are site-specific and phase-dependent. The general trends demonstrate responses producing decreased underfoot pressure at the site of stimulation.

**Conclusions:**

The responses to stimulation of discrete locations on the foot sole evoke a kind of “sensory steering” that may promote balance and maintenance of locomotion through the modulation of limb loading and foot placement. These results have implications for using sensory stimulation as a therapeutic modality during gait retraining (e.g. after stroke) as well as for footwear design and implementation of foot sole contact surfaces during gait.

## Background

Somatosensory feedback serves as a crucial means of communication between the environment and the central nervous system. There is a dynamic interaction between many different types of receptors in the lower limb including: nociceptors, muscle and skeletal mechanoreceptors, as well as cutaneous and subcutaneous mechanoreceptors [1,2]. Although all play relevant roles in locomotion, the activities of cutaneous and subcutaneous plantar reflex pathways are particularly important to ascertain in what way activation of cutaneous regions may differentially modulate pressure under the foot.

The role sensory feedback plays in sculpting human locomotion incorporates complex functional reflex modulation [3]. Reflexes have functional roles in locomotion that demonstrate context-dependent behavioural relevance. Reflex amplitudes (and thus the role of sensory input) are dependent upon task (standing vs. walking), stimulus intensity (nociceptive vs. non-nociceptive), and phase of movement (heel contact vs. stance to swing transition). The precise modulation of walking that is generated by cutaneous input from the foot depends upon the nerve that is stimulated [4,5]. Additionally, removal of the “normal” sensation from the skin of the foot alters muscle activation and gait mechanics [6]. Thus, cutaneous afferent feedback is suggested to assist in balance regulation and ensure proper foot placement during the stance phase [3,7,8].

There is a distinct nerve or location specificity of cutaneous reflexes. Activation of the foot dorsum, the lateral foot margin (sural nerve) and the plantar foot surface (tibial nerve) show differential responses [4,5,9-11]. In particular, receptors in the foot sole are the unseen sensory organs through which we perceive the ground during locomotion. Due to a high concentration of receptors including Meissner’s corpuscles, Merkel disk receptors, Ruffini endings, and Pacinian corpuscles, the glaborous skin of the plantar foot is sensitive to tactile input throughout the step cycle.

With electrical stimulation of the tibial nerve at tactile intensities, enhanced ankle dorsiflexion occurs at the stance to swing transition while ankle plantar flexion can be enhanced at swing to stance transition [4,10,12]. Non-nociceptive stimulation of the sural nerve during stance enhances dorsiflexion and eversion at the ankle in order to restore balance when on uneven terrain at the lateral foot surface and near the heel [11].

While non-noxious cutaneous reflexes have been studied for the three main nerve trunks innervating the dorsal and plantar foot, only recently have attempts to isolate specific tactile effects from regions of the foot sole been made [13,14]. These studies were restricted to sitting and standing conditions, and limited to forefoot medial, forefoot lateral, and heel stimulation but clearly showed that cutaneous reflexes evoked by stimulation of discrete foot sole regions produced topographical organized reflexes in human ankle muscles [13,14].

Forefoot stimulation produced inhibitory responses in the soleus (Sol) and medial gastrocnemius (MG), but excitatory responses in tibialis anterior (TA) muscles. Following heel stimulation, an opposite effect was evoked [14]. Systemic stimulations from the fifth toe to the heel on the lateral margin of the plantar foot demonstrated that the border of this Sol and TA reflex reversal occurred roughly around the middle of the foot sole, providing greater resolution in the fine sculpting of motor output than previously revealed by sural or tibial nerve stimulation.

It was also observed that stimulation at the lateral forefoot and heel evoked excitatory responses in peroneus longus (PL), but following medial forefoot stimulation an inhibitory response was evoked [13,14]. These results suggest tactile stimulation mimics a destabilization of posture and thus modulates PL responses to counteract uneven terrain through stabilization of the ankle joint [13,14].

An overarching principle in neuroscience, however, is that neural function is exquisitely task-dependent. As such we cannot predict with surety the pattern of reflexes evoked by foot sole stimulation during locomotion from data obtained during standing or sitting. The purpose of the current study was to examine cutaneous reflexes evoked by distinct plantar foot regions during locomotion. Additionally, we simultaneously look to determine the neuromechanical role of specific regions from the foot sole in shaping locomotor output and to initiate the process of developing a more detailed topographical view of the neuromechanical effects of cutaneous input from the foot during walking.

There were thus two main hypotheses tested in this study. Firstly, we hypothesized that stimulation at discrete skin locations on the foot sole would evoke topographically discrete cutaneous reflexes during walking. Secondly, we hypothesized that neural responses evoked in the form of cutaneous reflexes would have mechanical correlates detected as changes in force under the foot and kinematics of the stimulated leg.

## Methods

### Participants

Fourteen neurologically intact volunteers (8 females and 6 males) participated in the study. Participants were 30.3 ± 10 years old, with an average height of 174.4 ± 8.4 cm and weight of 73.7 ± 15.3 kg. Informed, written consent was obtained from all participants prior to the experiment under a protocol approved by the University of Victoria Human Research Ethics Committee and performed in accordance with the Declaration of Helsinki.

### Experimental protocol

**Figure 1:**
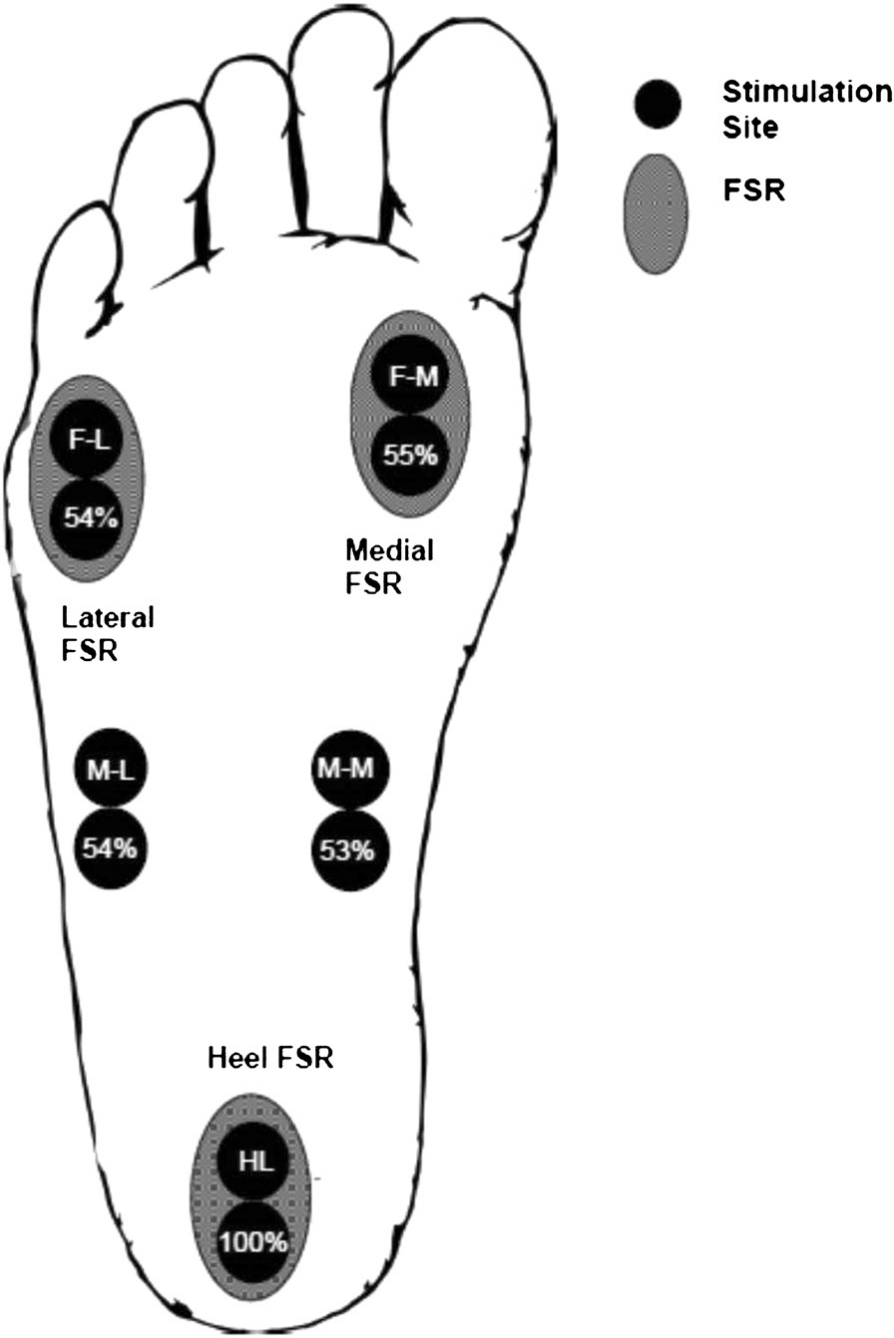
**Cartoon schematic illustrating the positions of the paired stimulation electrodes (solid circles) for the 5 sites: HL (S1), M-M (S2), M-L (S3), F-M (S4), and F-L (S5).** The position of the force-sensing resistors (FSRs) are shown as the hatched ovals at heel (F1), medial (F2), and lateral (F3) regions. Abbreviations: M-M = mid-foot medial, M-L = mid-foot lateral, F-M = forefoot medial, F-L = forefoot lateral.

To improve procedural standardization and consistency, all participants were provided and fitted with the same make and model of running shoe with flexible foot sole (Nike Free 3.0 V4). The sipes (grid like horizontal cuts along rubber sole) enhanced flexibility, while the seamless upper and stretchy inner-sleeve provided a sock-like snug fit, both of which were useful in evaluating plantar cutaneous reflex effects during locomotion. The right shoe was then equipped with an insole containing five paired stimulating electrodes and three force sensing resistors (FSRs), as shown in Figure 1.

Participants walked on a motorized treadmill (Woodway USA, Waukesha, WI) at a self-selected, comfortable walking pace (2.1 ± 0.5 mph) that remained constant throughout the experiment. During each trial (average duration 315.6 ± 11.4 seconds), participants walked continuously while electrical stimulation was delivered to one of the five stimulation sites. In total, five separate trials were performed in order to obtain responses to stimuli for each regions stimulated. Prior to the experiment, the order of presentation for each of the five trials was randomized.

### Cutaneous stimulation

**Figure 2:**
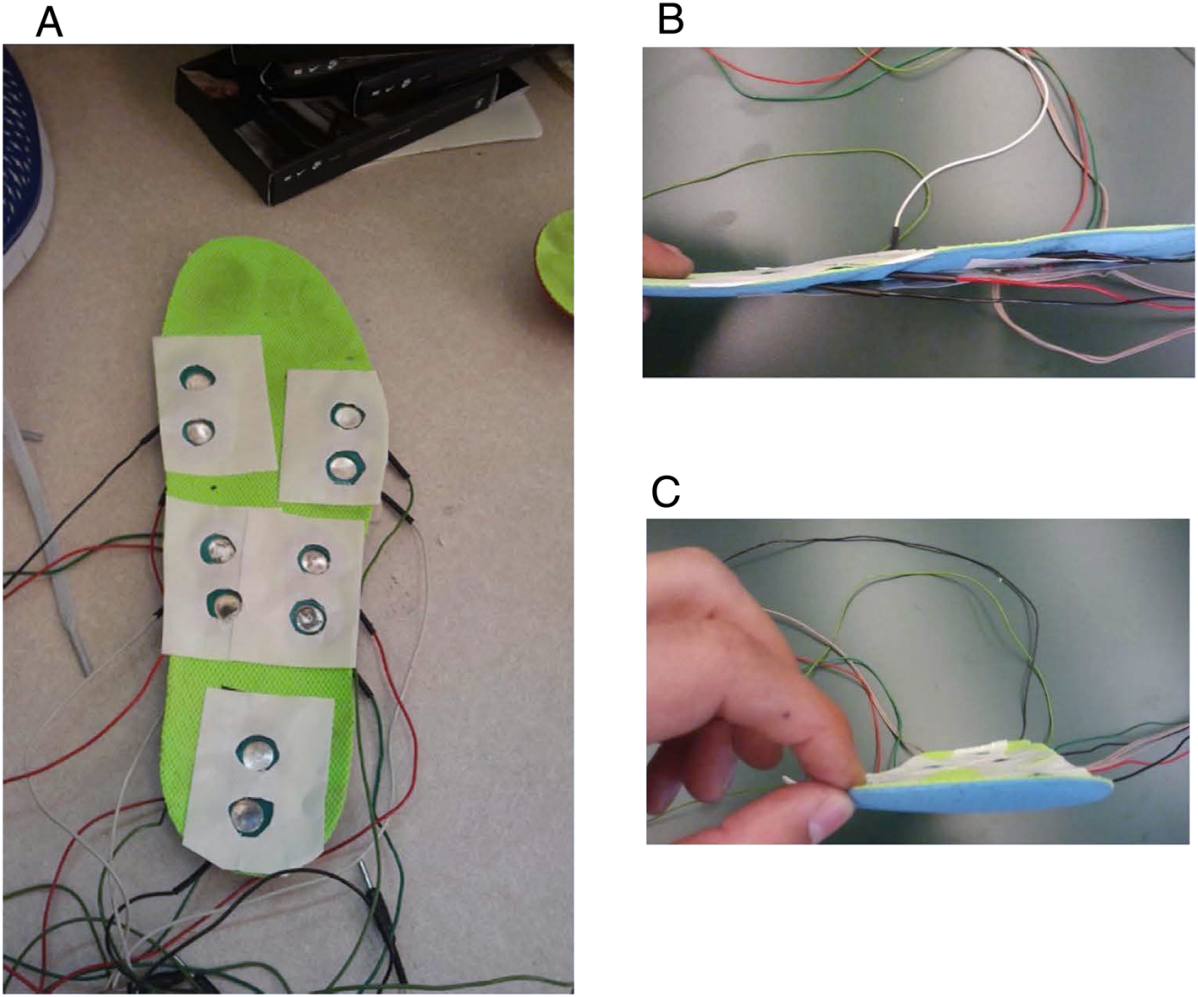
**Photographs showing the configuration of the stimulating electrode array in the sock liner. A**. the electrode pairs cut-out from the sock liner and overlaid with 2 sided tape; and profile of the integrated sock liner with electrode pairs and FSRs shown from the medial side **(B)** and from the front **(C)**.

Transcutaneous electrical stimulation was delivered separately and in random order to five sites on the sole of the right foot. These sites were: calcaneous (HL); medial aspect of the transverse arch of the foot (M-M); proximal end of the fifth metatarsal (M-L); head of the first metatarsal (F-M); and, distal end of the fifth metatarsal (F-L) (Figure 1).Stimulation was provided by a Digitimer Constant Current High Voltage Stimulator (Model DS7AH) with trains of 5 × 1.0 ms pulses at 300 Hz. Stimulation to the bottom of the foot was delivered via paired surface electrodes (cathode distal) (Narco Bio-systems INC, Houston, TX) placed within the insole of the right shoe to minimize undesired sensation, discomfort, and movement of the electrodes. Holes were cut and bevelled at the locations indicated in Figure 2. Figure 2a shows the relative position of the 5 stimulus sites on one of the sock liners. Figures 2b and c show the medial and lateral profiles, respectively, for the sock liner when viewed from the side. The plastic electrodes were then inserted through these holes and adhered with tape to provide a flush fit against the bottom of the sock liner. Two sided tape (cut with appropriate holes for each electrode) was then affixed over each electrode site and attached tightly to the foot sole.

Next, the electrodes were filled with electrode gel (Parker Laboratories, INC, Orange, NJ) to improve electrical conduction with the skin surface of the bare foot of each Participant. During each trial, a total of 160 randomly-timed stimulations (1–3 seconds) were delivered throughout the step cycle.

### Stimulation intensity

Immediately prior to each trial, perceptual threshold (PT) was determined for each stimulation site. PT was defined as the stimulus intensity found to evoke detectable tactile sensation at the lowest intensity possible. Participants remained standing, while stimulation intensities were gradually decreased by the researchers until the participant could barely discern the stimulus (identified as PT). The stimulation intensity delivered to the sole of the foot was set to approximately three times PT. The actual stimulator output needed to reach PT was the same at all sites except for the heel where it was ~ twice as high. (The actual percentage of stimulator output needed to achieve PT in all sites with HL as the reference is shown within the lower circles for stimulation sites in Figure 1). The stimulation intensity of 3 × PT was chosen in order to evoke a non-noxious cutaneous sensation during each trial by activating cutaneous afferents immediately under the electrodes and to provide the same relative activation at all stimulation sites.

### Electromyography

Once the skin was cleaned with alcohol wipes, disposable surface electrodes were placed on the skin over muscles in the upper and lower leg and shoulder. All EMG recordings were ipsilateral to the site of stimulation (right side). Muscles recorded from included TA, MG, PL, vastus lateralis (VL), biceps femoris (BF), gracilis (GR), gluteus medius (GM) and posterior deltoid (PD). Ground electrodes were placed over electrically neutral tissue. EMG signals were amplified at 5000 times and filtered from 100–300 Hz (Grass P511, Astro-Med Grass Inc.).

### Mechanical measures: force sensing resistors and goniometry

Force sensing resistors (FSRs) were firmly attached to the insole of the participant’s right shoe. The forces produced at the foot-insole interface during the step cycle were recorded by three custom-made FSRs located at anatomically distinct landmarks: calcaneous (heel), head of the first metatarsal (medial), and distal end of the fifth metatarsal (lateral) (Refer to Figure 1). These signals were also used to establish step cycle parameters (such as heel contact and stance to swing transition) based on methods previously described [4]. Throughout the experiment, force signals were pre-amplified, and sent directly to the computer system. FSR signals from both unperturbed steps, serving as the control, and perturbed steps were recorded in volts and saved for later analysis.

Angular positions of the hip (flexion/extension), knee (flexion/extension), and ankle (inversion/eversion and dorsi/plantarflexion) were measured with electrogoniometers (Biometrics Ltd., Gwent, UK).

### Data analysis

**Figure 3:**
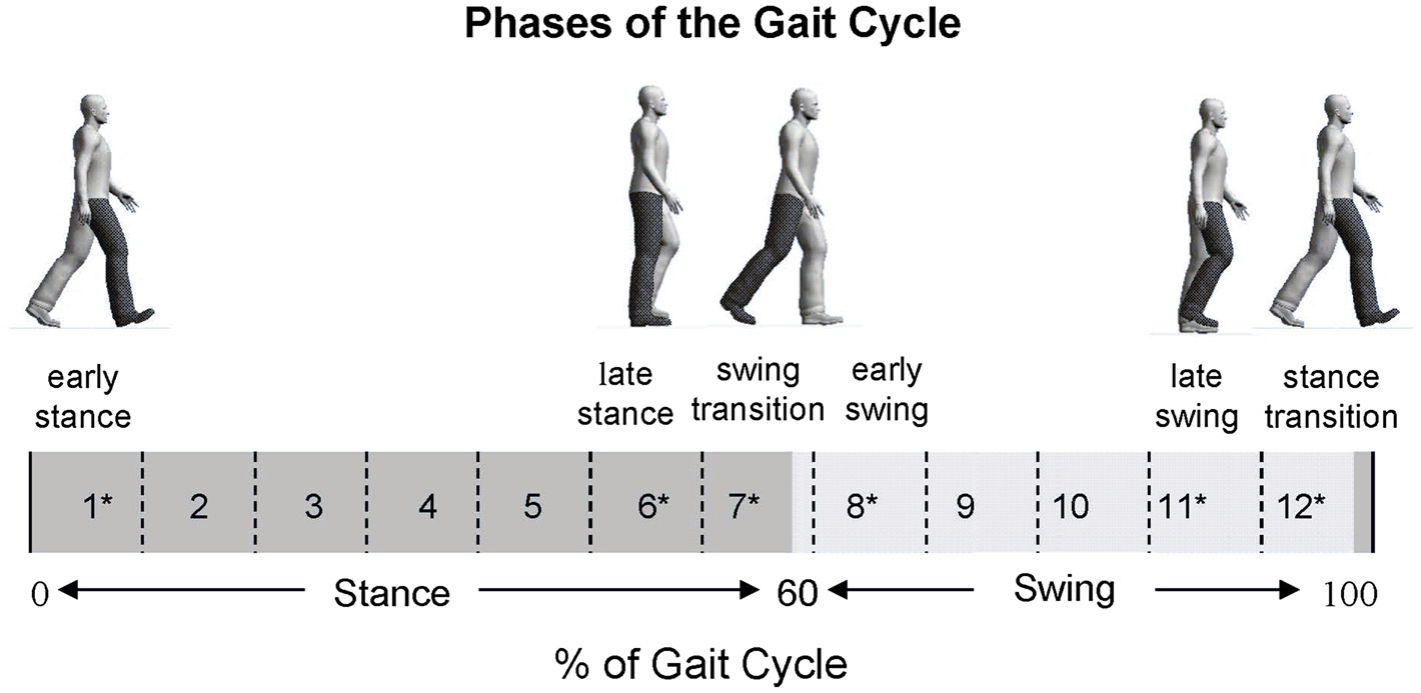
Schematic illustration of the walking cycle along with phase numbering for analysis and highlights of specifically targeted parts of stepping.

As with our other similar studies ([4,15-17]), all EMG, kinetic and kinematic data were sampled at 1000 Hz with a 12 bit A/D converter connected to a PC running custom-written LabVIEW (National Instruments Corp., Austin, Texas, USA) acquisition software and stored on hard disk for off-line analysis. Offline data were analysed using custom written software MATLAB (MathWorks, Inc., Natick, MA, USA). Custom-written software programs were used to separate the step cycle into 12 separate phases, beginning with heel contact and ending with the subsequent heel contact at the swing to stance transition. Using the FSR data, all responses for all data for each stimulus condition occurring in the same phase of the step cycle (n = ~10–20 responses per phase; See Figure 3 for schematic illustration of the phases of walking) were aligned to stimulus delivery and averaged together. Assignment to phase of step cycle was based on the time of the stimulation delivery in the data sweep. Averages from the same phase of walking during unstimulated cycles (“control” EMG n = ~50–60 per phase) were then subtracted from each of the corresponding 12 averages after stimulation yielding subtracted traces of reflex EMG, and stimulation induced changes in kinematic and kinetics. For evaluation of reflex effects the subtracted data traces were analyzed in all instances.

Cutaneous reflexes were determined as the average cumulative reflex activity occurring 150 ms after stimulation (ACRE150). This “net reflex” has been shown to be well correlated with kinematic responses in the legs [4,11,12,18] and arms [19]. This measure involved calculating a subtracted reflex (see above) then cumulatively summing the signal over a post-stimulus period of 150 ms. The summed value was then divided by the time interval of integration to measure an overall reflex effect. Net reflex values were normalized to peak control EMG amplitude.

Mechanical data were low pass filtered at 20 Hz using a dual-pass third order Butterworth filter. Stimulated data were subtracted from unstimulated data to yield a reflex trace. Mechanical reflex changes were analyzed within a 140–220 ms window post stimulus [4,19]. Responses were considered significant if they exceeded a 2-SD band of the mean value of the subtracted prestimulus level of the ongoing mechanical parameter at each phase.

### Statistics

All statistical analyses were completed using SPSS version 18 (Chicago, IL). In all instances, analysis was conducted on averaged data from each part of the step cycle for each Participant. Each data set was analyzed separately, as was each phase of walking, in order to determine if the site of stimulation at a specific location on the foot sole and during a particular part of the step cycle had a discernible effect.

To test our first hypothesis that stimulation at discrete locations on the foot sole would evoke topographically distinct cutaneous reflexes, the initial approach for all data was to conduct 12 omnibus 12 (Phase) × 5 (Sites of stimulation) repeated measures ANOVAs. Note that all significant differences indicated on the figures also showed a significant 1× 5 ANOVA main effect. Fisher’s LSD post hoc tests were used to determine site specific differences.

Based on the critical function of somatosensory feedback particularly at transition points and less stable phases of walking [3] we predicted enhanced effects from stimulation sites at early stance (phase 1; see Figure 3), late stance (phase 6), the swing transition (phase 7), early swing (phase 8), late swing (phase 11), and the stance transition (phase 12). Thus, using an approach applied in prior work [16,20], we subsequently performed planned comparisons between stimulation sites at these phases (also noted with asterisks on Figure 3). On all data figures across phases of walking, these phases are identified with black borders around the plots.

All statistical tests were 2-tailed and significance was accepted at *p* < 0.05. Thus, all data described in the following text or shown in the accompanying figures and described as “significant” or indicated with an * were determined as a main effect or interaction from the Omnibus and from the RM ANOVA and tested with Fisher’s LSD post hoc test at the level of *p* < 0.05.

## Results

### Cutaneous reflexes

**Figure 4:**
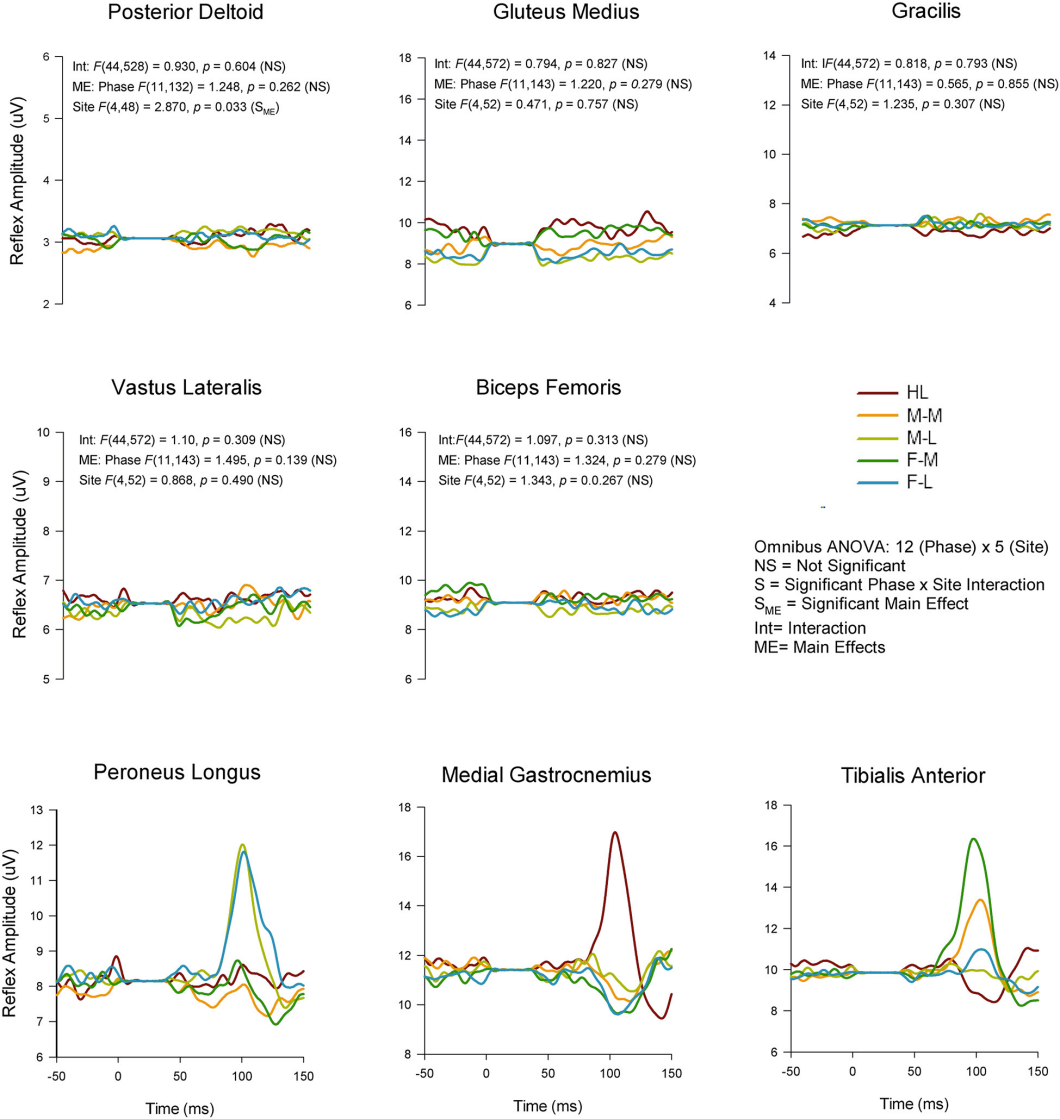
**Grand average reflex traces taken across the entire walking cycle for all 5 stimulation conditions across all participants in all 8 muscles.** Note that presentation of the data in this format shows nothing about phase-modulation since all stimulation irrespective of phase are averaged together. This presentation emphasizes the major trends in the reflexes but can obscure details such as phase-modulation. Abbreviations: HL (heel), M-M (midfoot medial), M-L (midfoot lateral), F-M (forefoot medial), F-L (forefoot lateral). The stimulus artefact beginning at time 0 has been blanked out and replaced by a flat line. The outcomes from interaction terms in the omnibus ANOVA are listed in the panel for those muscles not subjected to further analysis.

To view any evoked reflexes in all muscles studied we present the phase-independently averaged data in Figure 4. The data traces represented are averages across all Participants from all stimuli given across the entire step cycle for each Participant. This process reveals strong reflex effects but can obscure phase-modulation and other subtleties. This figure is useful, though, for representing the general trends in the evoked responses.

**Figure 5:**
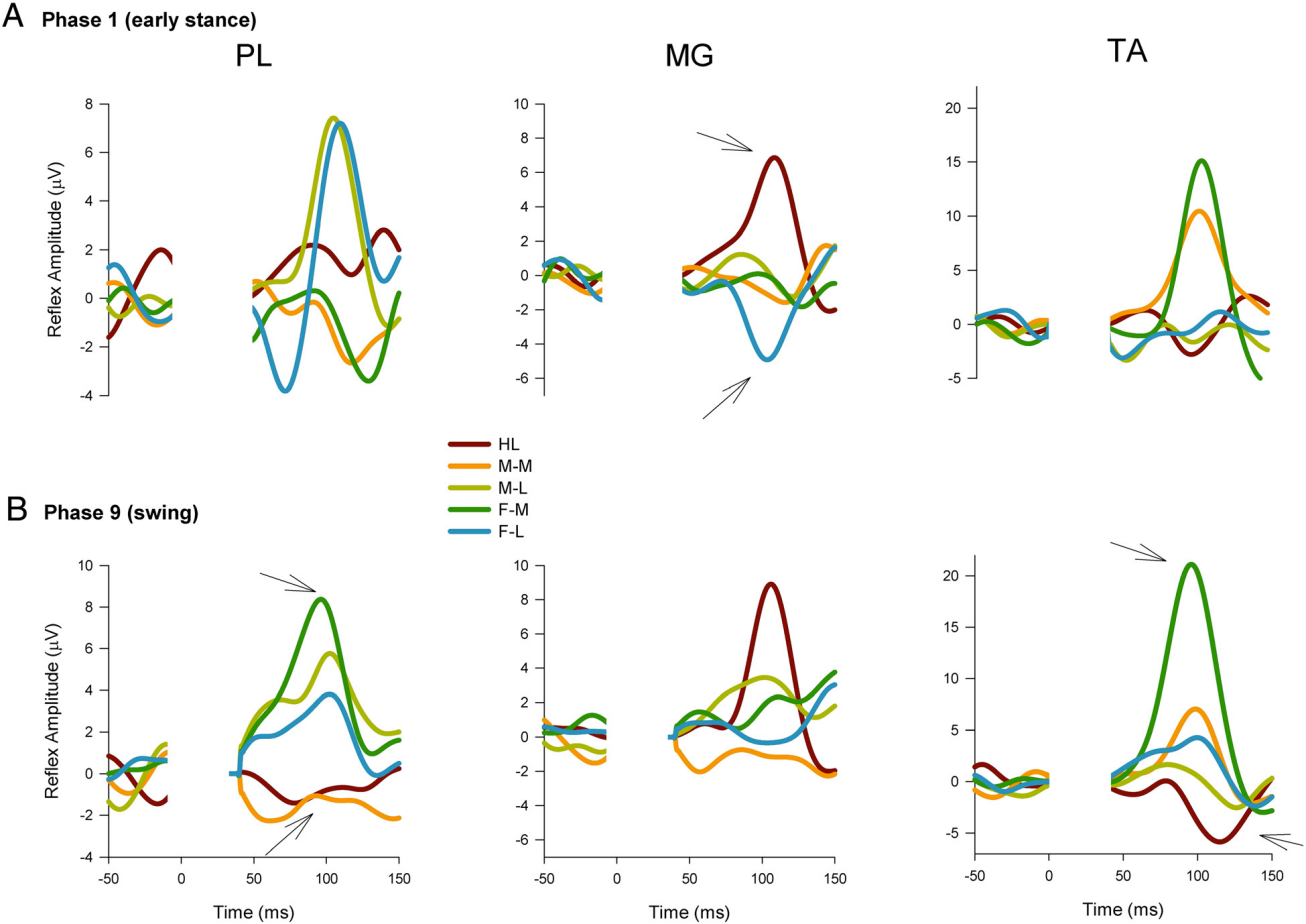
**Grand average reflex traces for all 5 stimulation conditions across all Participants (n = 16) in PL, MG, and TA muscles at specific walking phases. A)** early stance (phase 1), and **B)** swing (phase 9). Abbreviations: PL = peroneus longus, MG = medial gastrocnemius, TA = tibialis anterior. Other abbreviations as in Figure 4. Note that the stimulus artefact has been removed from all traces and replaced by a flat line extending from time 0 to ~50 ms post-stimulation.

**Figure 6:**
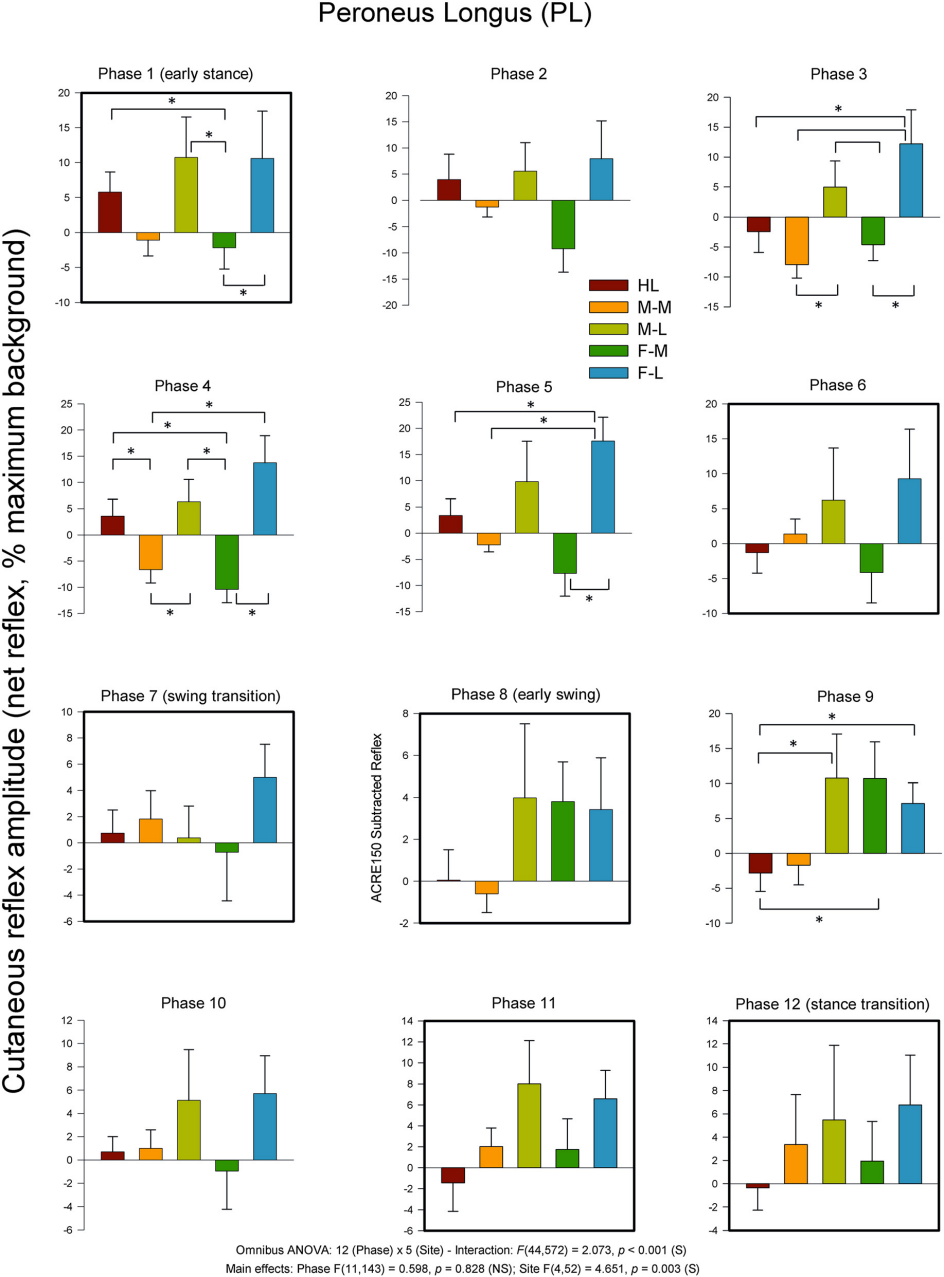
**Average quantified net (ACRE**_**150**_**) cutaneous reflexes across all 12 phases of the step cycle for ankle plantarflexor and evertor muscle peroneus longus.** Data are percentages normalized to maximum background EMG measured across all phases of walking. Negative values indicate overall suppression and positive values overall facilitation of muscle activity. There were significant main effects for phase and stimulus region as well as a phase × region interaction. Phases of walking analyzed with planned comparisons are indicated by black borders. *indicates statistical differences at p < 0.05 between stimulation conditions within a phase.

When looking at specific phases of walking, differences in evoked cutaneous reflexes were found extensively across the walking cycle in lower leg muscles PL, MG, and TA. In the other muscles examined, differences based upon site of stimulation were not typically observed. Taking into account PD, BF, VL, and GM, only 2 phases of walking (one in VL, one in PD; out of a possible 48 phases across muscles) showed a main effect for stimulation site. Thus these data are not discussed further below and the focus remains on the 3 lower leg muscles acting predominately at the ankle.On Figure 4 are also shown the results of main effects and interaction terms from the omnibus ANOVA for each that did not show site-specific effects. The data shown in Figure 4 clearly illustrate that the main outcomes of regional foot sole stimulation emerged in PL, MG, and TA muscles (statistical details found on subsequent figures).Grand averages taken across all participants for each stimulation condition for PL, MG, and TA muscles are shown in Figure 5 row A for Phase 1 (early stance) and for Phase 9 (swing) in row B. Three main observations are demonstrated in this Figure. First, comparison across stimulation conditions within a muscle show the site-dependence of the reflexes. For example, reflexes in MG at Phase 1 show clear antagonism with facilitation in the HL condition and suppression for F-L (see arrows). Similarly, reflexes at Phase 9 for PL muscle in the F-M and MF Med show opposite effects as do HL and F-M for TA muscle (again see arrows on Figure). Second, comparison across muscles within a stimulation condition show that the reflex effects are differentially specified to muscle. For example, compare responses for F-M in PL (facilitation), MG (no effect) and TA (facilitation) for F-M at Phase 9. Third, comparison within a muscle and stimulation site across phases shows clear phase dependence. For example, compare F-M in PL muscle from Phase 1 (suppression) to phase 9 (facilitation).Characteristics evident in the reflex traces are illustrated in the quantified PL net reflexes shown in Figure 6. As indicated on the figure, PL reflexes showed a significant main effect for phase and site and a phase × site interaction. Site-dependence can be assessed in several ways including statistical differences across stimulation condition and sign of the evoked responses. In PL muscle, functional dorsiflexor and ankle invertor, significant site-dependence of foot sole stimulation was found in 5 of the 12 step cycle phases: four during stance (phases 1,3–5) and one during swing (phase 9). During stance, a key observation is that F-M and F-L were always (phases 1,3,4 &5) significantly different from each other and M-M and M-L differed from each other in mid-stance (phases 3 and 4). During swing (phase 9) the distal sites (M-L, F-M, F-L) differed from more proximal sites (HL & M-M).

Additionally, when considering the sign of the responses, there were clear site-dependent effects that can be seen in the Figure. Notably, facilitation was evoked in PL for all 12 phases of the step cycle when stimulation was applied to the lateral region of the foot (M-L and F-L). Heel stimulation produced facilitation in 7 phases and suppression in 5 whereas the opposite distribution was found at M-M with 5 phases with facilitation and 7 with suppression. F-M had 4 phases with facilitation and 8 with suppression.

**Figure 7:**
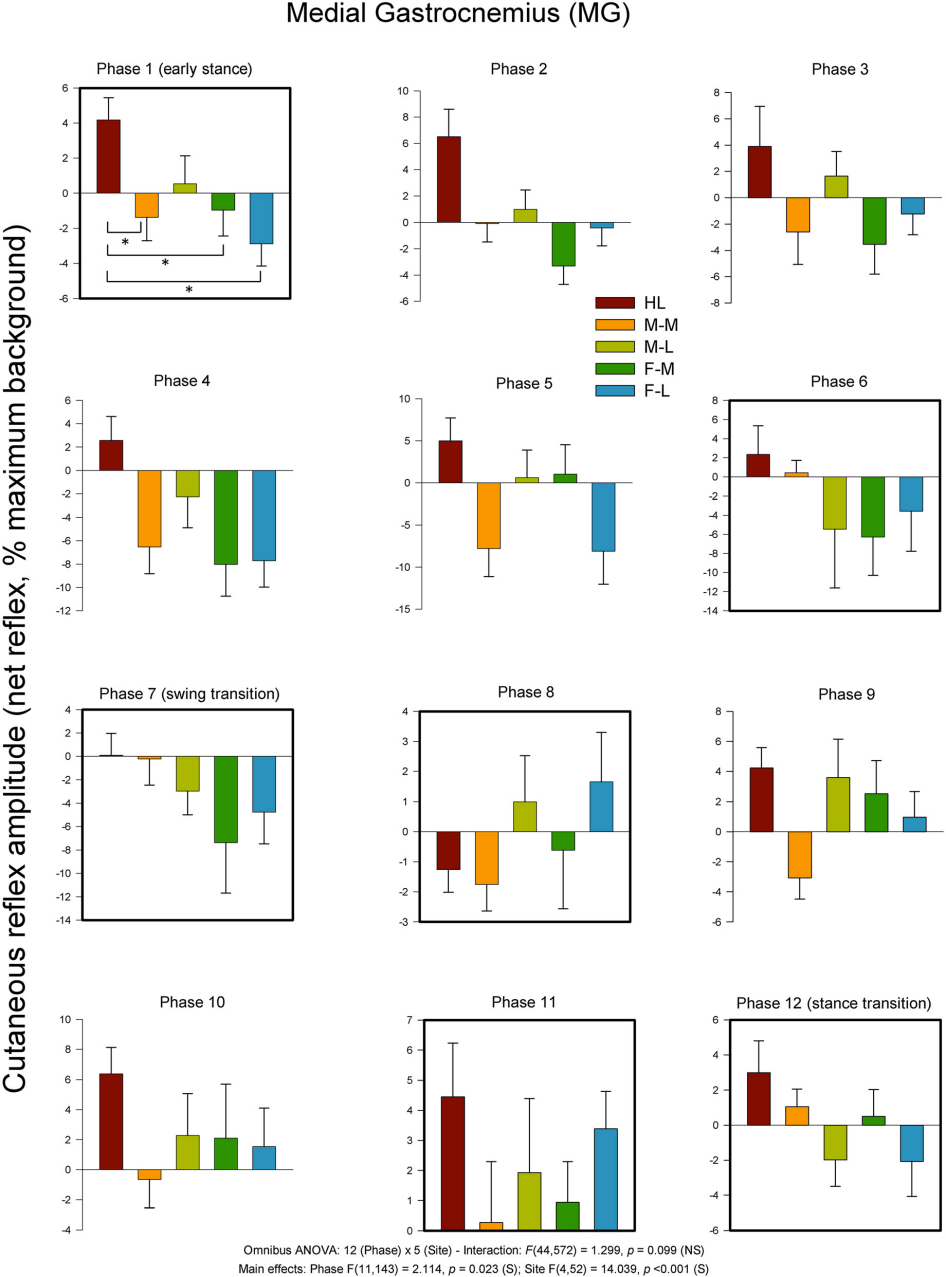
**Average quantified net (ACRE**_**150**_**) cutaneous reflexes across all 12 phases of the step cycle for ankle plantarflexor and evertor muscle medial gastrocnemius.** Data are percentages normalized to maximum background EMG detected across all phases of walking. Negative values indicate overall suppression and positive values overall facilitation of muscle activity. There were significant main effects for phase and stimulus region. Phases of walking analyzed with planned comparisons are indicated by black borders. *indicates statistical differences at p < 0.05 between stimulation conditions within a phase.

MG muscle, is a strong plantarflexor and ankle evertor. Within the Achilles tendon, the fibers of the MG make their way to the lateral portion of the calcaneus as originally described by Parsons in 1894 [21] and discussed in Zehr et al. (1998) [11]. As shown on Figure 7, for reflexes in MG there was a significant main effect for phase and stimulation site, but no significant interaction. Phase 1 (early stance) showed significant differences between nerve stimulation sites. A main observation for MG muscle was that the HL site typically differed from at least one other stimulation site, particularly during stance.

**Figure 8:**
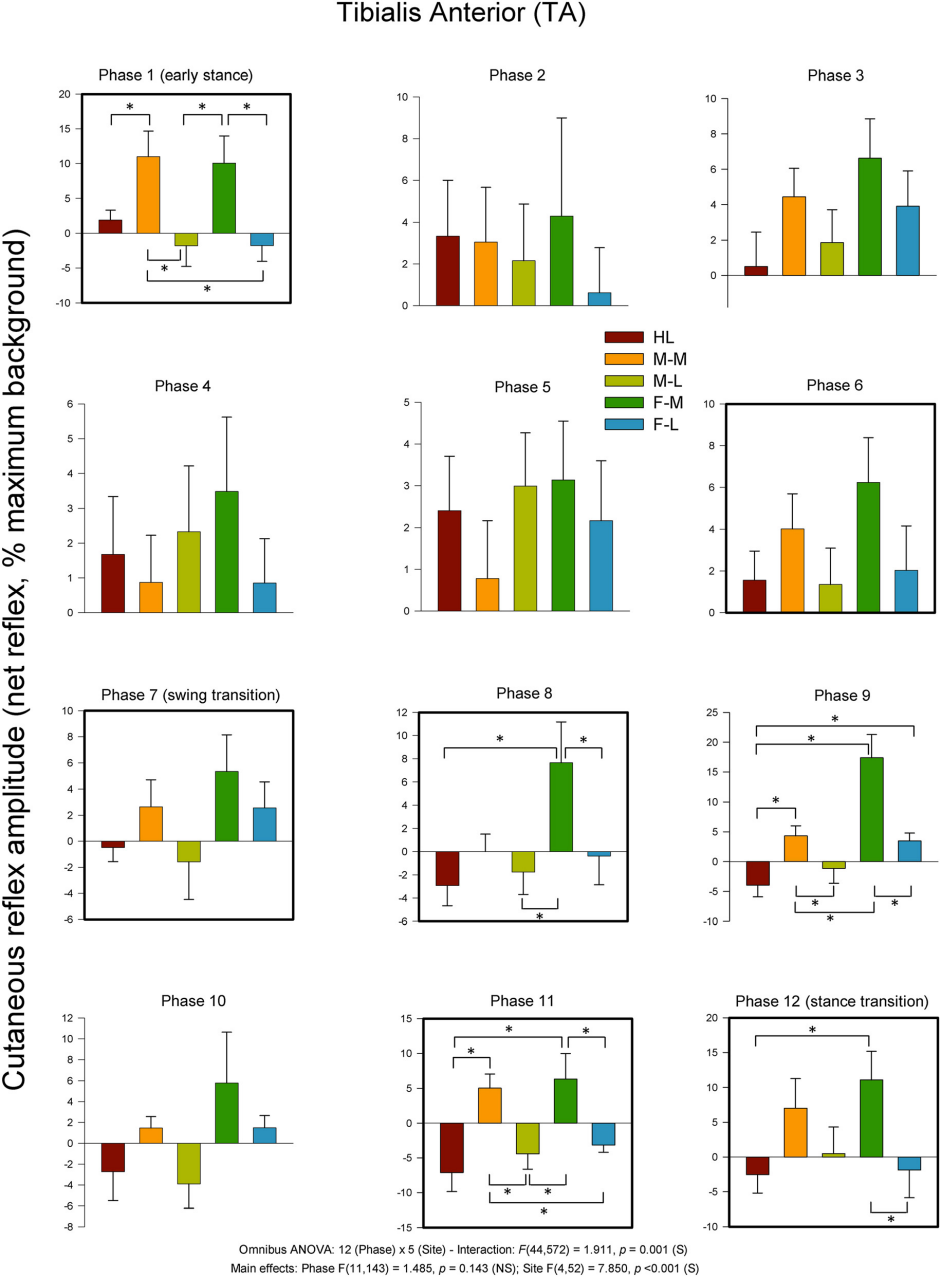
**Average quantified net (ACRE**_**150**_**) cutaneous reflexes across all 12 phases of the step cycle for ankle dorsiflexor and invertor muscle tibialis anterior.** Data are percentages normalized to maximum background EMG detected across all phases of walking. Negative values indicate overall suppression and positive values overall facilitation of muscle activity. There were significant main effects for phase and stimulus region as well as a phase × region interaction. Phases of walking analyzed with planned comparisons are indicated by black borders. *indicates statistical differences at p < 0.05 between stimulation conditions within a phase.

As for the sign of the evoked responses, the most notable effect was 11 phases with facilitation for HL and only one with suppression. The medial sites showed mostly suppression (M-M = 9 and F-M = 7). There were similar effects for the lateral sites with F-L (n = 8) and F-M (n = 7) showing largely suppression.In TA muscle (see Figure 8), functional dorsiflexor and ankle invertor, cutaneous reflexes showed significant main effects for phase and site and a significant phase × site interaction. Five of the step cycle phases showed significant differences across nerve stimulation sites. Three were during swing (8, 9 & 11), and 2 included the transition to stance (phase 12) and early stance (phase 1) itself. A main observation during swing phases 8, 9 and 11 was that F-M significantly differed from most other sites except M-M. Stimulation at the medial sites of M-M ad F-M produced facilitation of TA muscle at swing to stance transition (Phase 12).

In terms of the sign of the responses, for the medial stimulation sites, facilitation dominated (M-M = 12 and F-M = 12) all the phases of walking. Mixed results were seen at the HL (n = 6) and M-L (n = 6) and F-L had 8 phases with facilitation.

### Kinetics

#### Heel FSR

**Figure 9:**
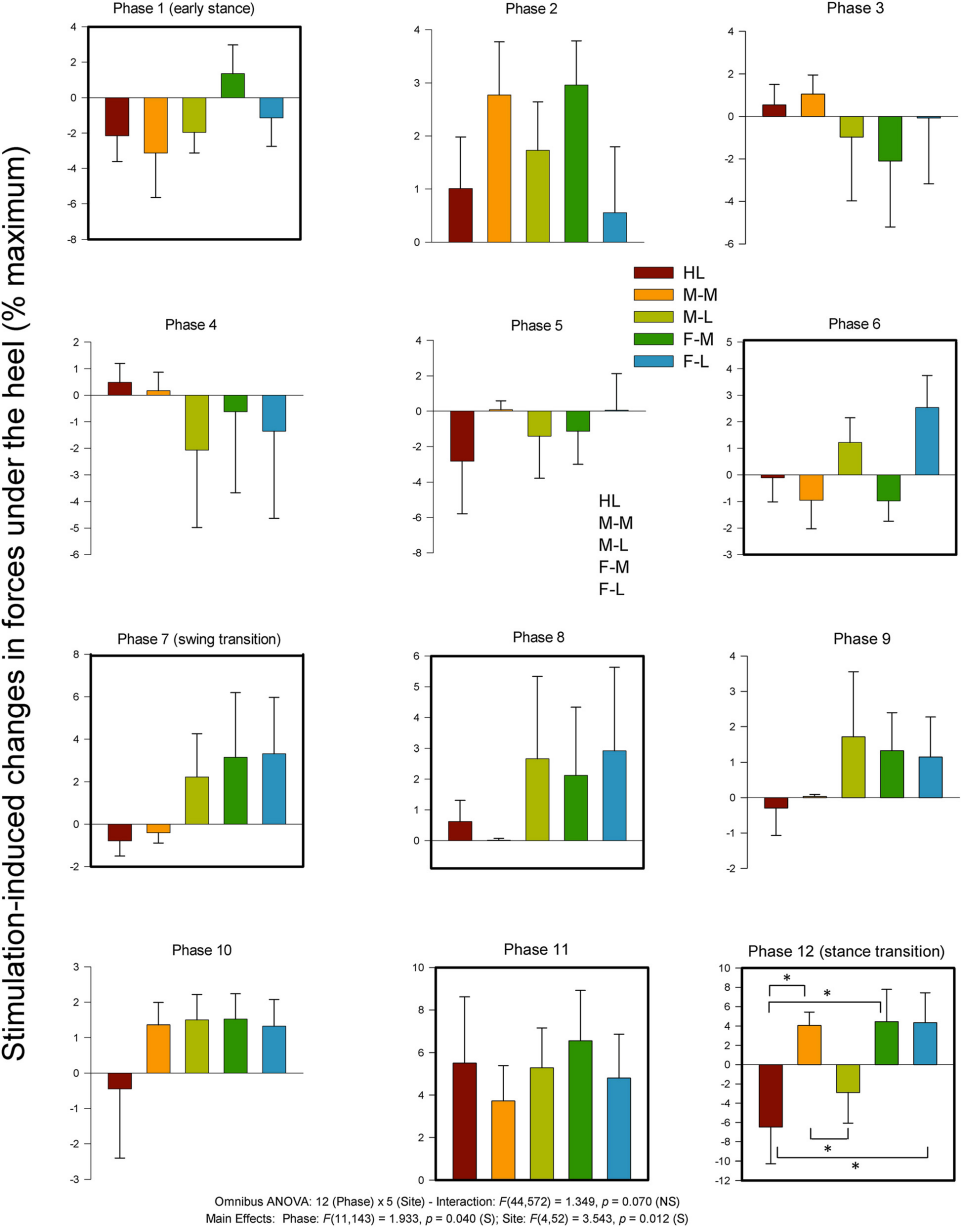
**Stimulation-induced average changes in forces under the foot detected by FSRs at the heel.** Data are percentages normalized to maximum FSR load detected in the stance phase of walking. Phases of walking analyzed with planned comparisons are indicated by black borders. *indicates statistical differences at p < 0.05 between stimulation conditions within a phase.

Statistical analysis of heel FSR data revealed main effects for phase and site and a significant phase × site interaction. Data from the heel FSR along with results from the statistical tests are plotted in Figure 9. During stance HL stimulation tended to produce reduction in force at the heel FSR. At the stance transition (phase 12) both forefoot sites and the M-L were significantly increased compared with HL stimulation.

#### Medial FSR

**Figure 10:**
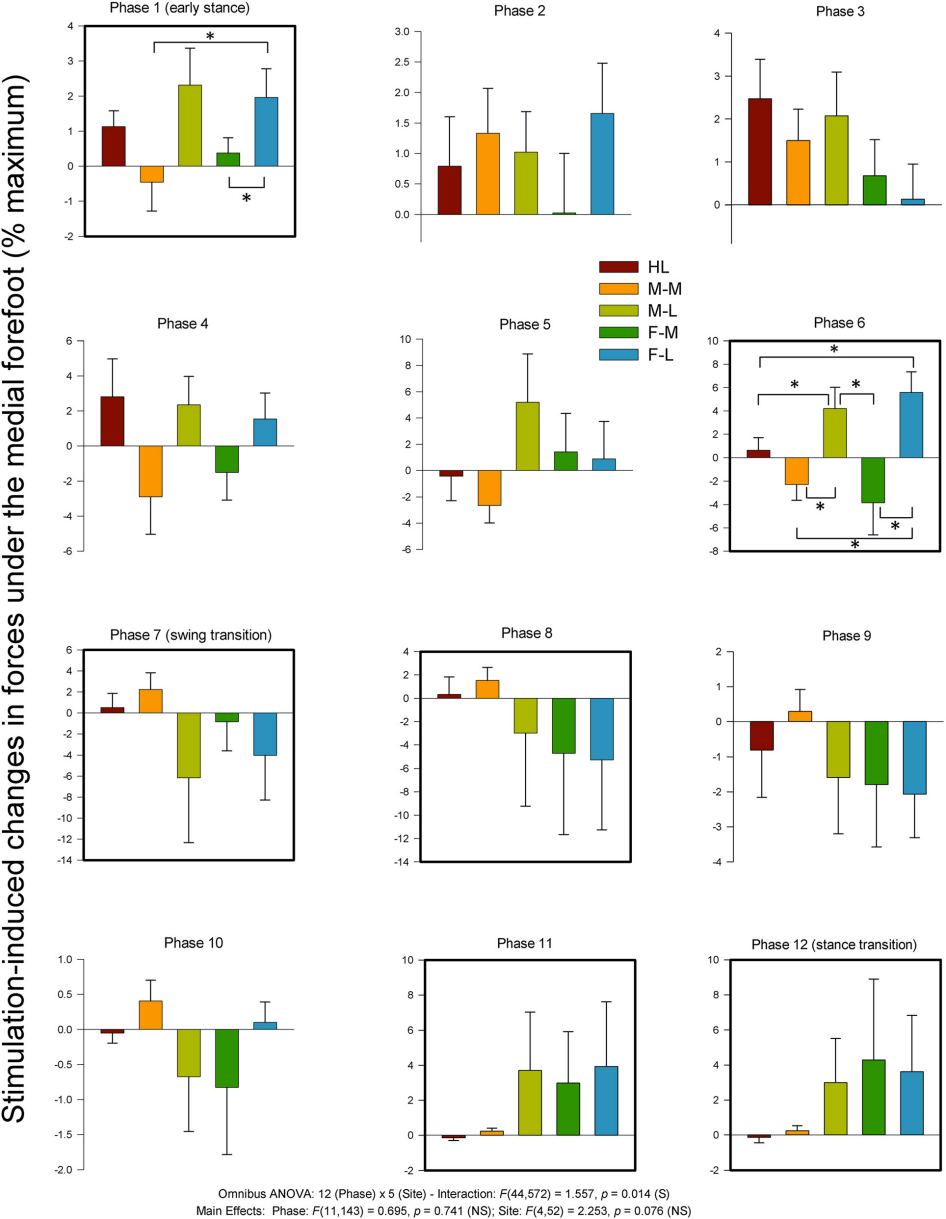
**Stimulation-induced average changes in forces under the foot detected by FSRs at the medial foot.** Data are percentages normalized to maximum FSR load detected in the stance phase of walking. Phases of walking analyzed with planned comparisons are indicated by black borders. *indicates statistical differences at p < 0.05 between stimulation conditions within a phase.

Statistical analysis of medial FSR data revealed main effects for phase and site and a significant phase × site interaction. Data from the medial FSR along with summary results from the statistical tests are plotted in Figure 10. Medial FSR showed site dependence at early (phase 1) and late stance (phase 6). Generally, lateral stimulation sites (M-L & F-L) tended to produce increases in force detected at the medial FSR during stance.

#### Lateral FSR

**Figure 11:**
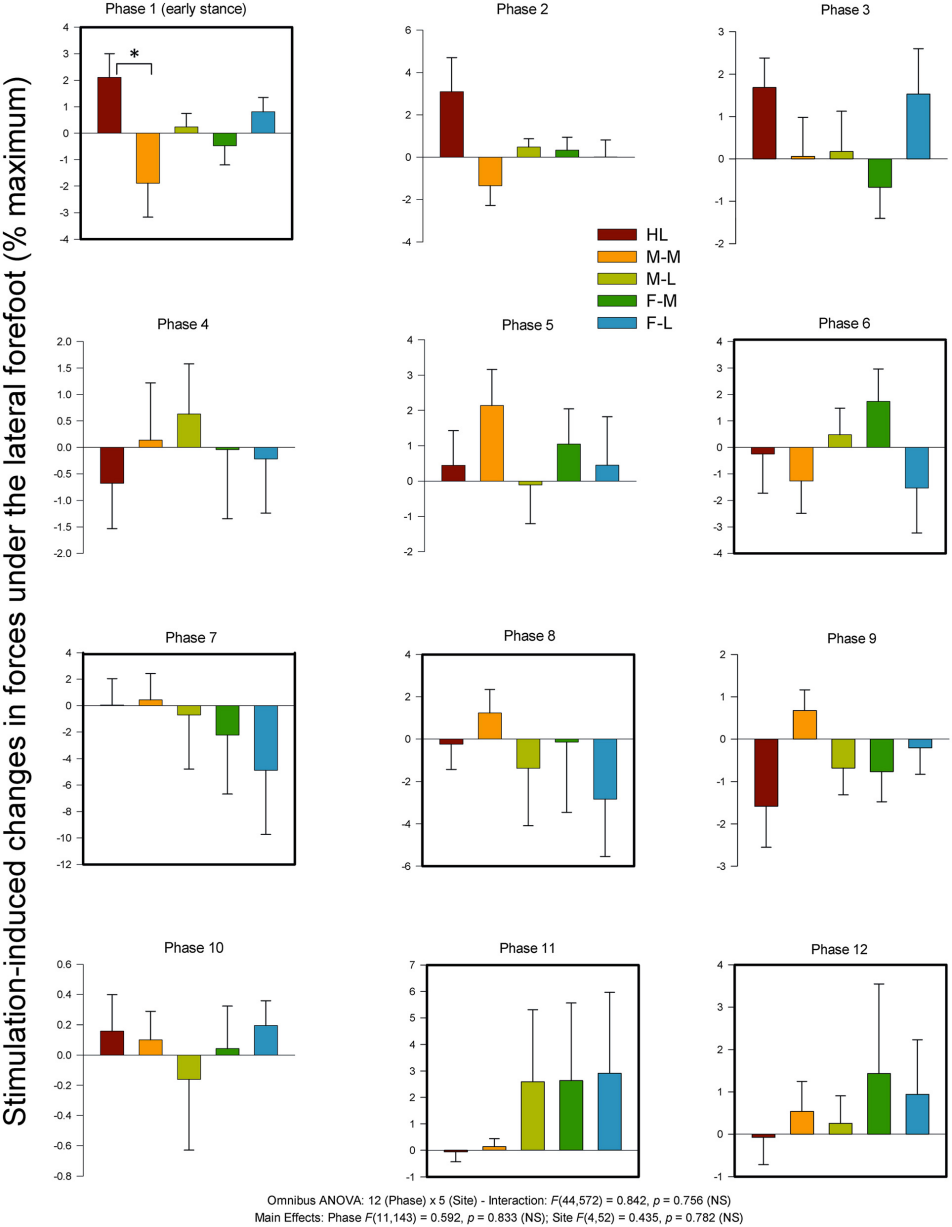
**Stimulation-induced average changes in forces under the foot detected by FSRs at the lateral margin.** Data are percentages normalized to maximum FSR load detected in the stance phase of walking. Phases of walking analyzed with planned comparisons are indicated by black borders. *indicates statistical differences at p < 0.05 between stimulation conditions within a phase.

Statistical analysis of lateral FSR data revealed no main effects for phase and site nor a significant phase × interaction. Data from the lateral FSR along with results from the statistical tests are plotted in Figure 11. Generally, medial stimulation sites M-M (phases 1 & 6) and F-M (phase 6) tended to produce reduced forces at the lateral FSR. The lateral FSR showed site dependence at early stance (phase 1) where medial stimulation at site M-M (reduction) and HL (increase) showed opposite effects at the lateral FSR.

#### Kinematics

As with the EMG data for the upper leg, there were no differences across stimulation conditions for knee or hip kinematics and these data are not plotted.

#### Ankle inversion and eversion

**Figure 12:**
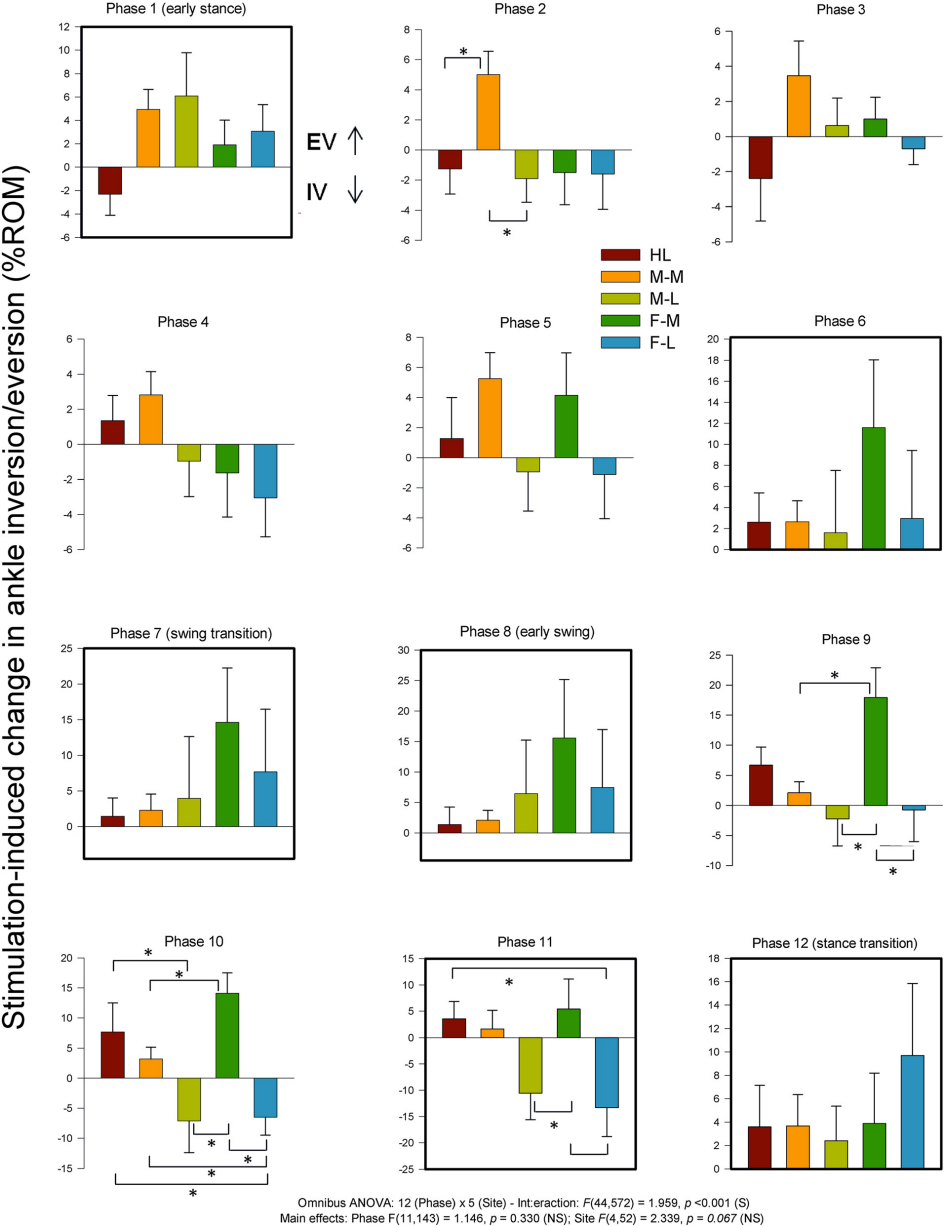
**Stimulation-induced average changes in ankle joint kinematics for inversion/eversion (eversion = up).** Data are percentages normalized to maximum range of motion across all phases of walking. Phases of walking analyzed with planned comparisons are indicated by black borders. *indicates statistical differences at p < 0.05 between stimulation conditions within a phase.

Ankle kinematics for inversion-eversion showed significant main effects for phase and site and a significant phase × site interaction. Data for stimulus-induced changes in ankle inversion-eversion across the step cycle along with results from the statistical tests are plotted in Figure 12. Significant site-dependent changes in kinematics were detected at the ankle for inversion and eversion during stance (phase 2) and throughout swing (phases 9–11). During stance, M-M stimulation reduced eversion and statistically differed from both HL and from M-L. During swing, a common feature for In/Ev was that FF Med differed from most other conditions. Stimulation at lateral sites (M-L and F-L) produced opposite responses (reduced inversion) to that seen with stimulation of medial sites (M-M and F-M; reduced eversion).

#### Ankle plantar and dorsiflexion

**Figure 13:**
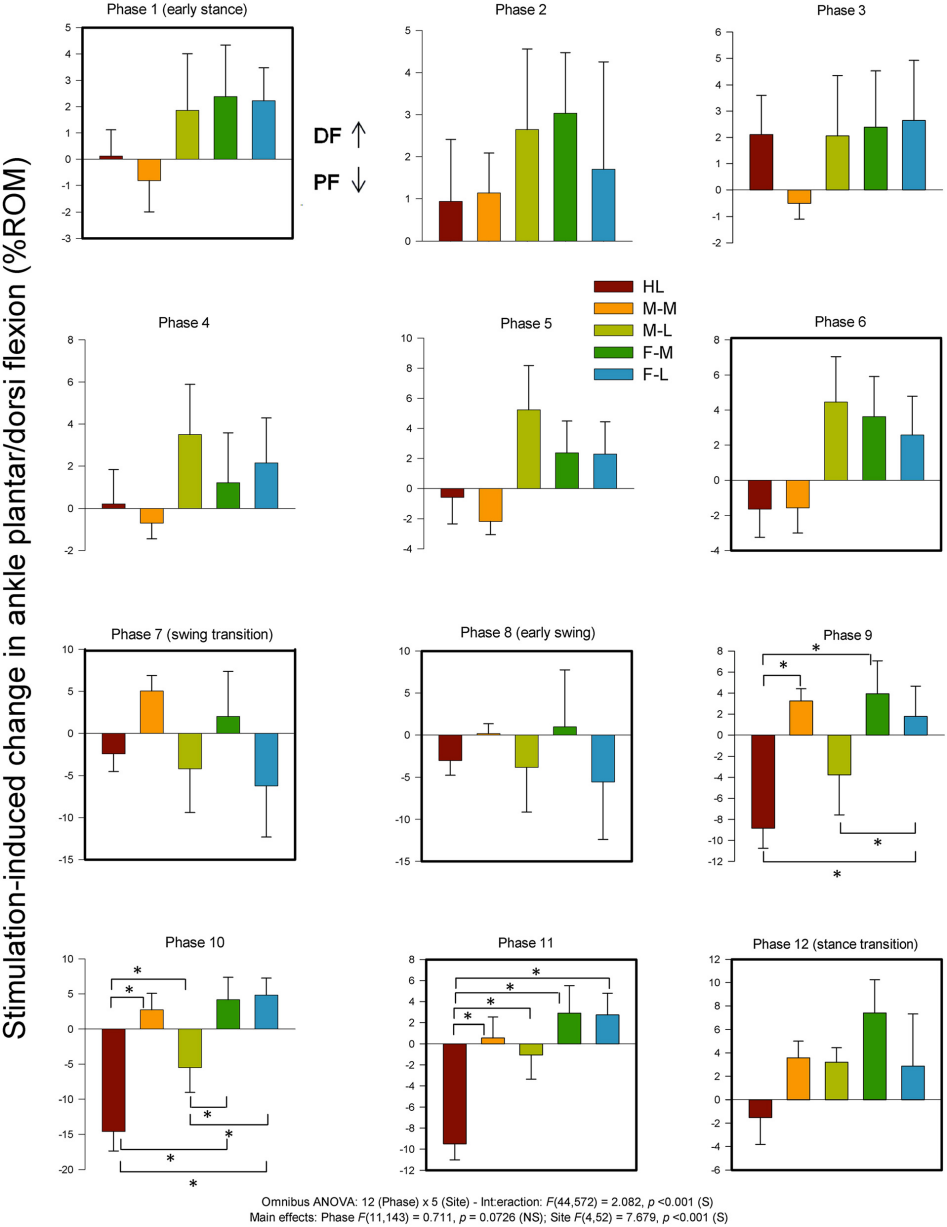
**Stimulation-induced average changes in ankle joint kinematics for dorsiflexion and plantarflexion (dorsiflexion = up).** Data are percentages normalized to maximum range of motion across all phases of walking. Phases of walking analyzed with planned comparisons are indicated by black borders. *indicates statistical differences at p < 0.05 between stimulation conditions within a phase.

Statistical analysis of ankle plantar and dorsiflexion revealed main effects for phase and site and a significant phase × site interaction. Data for plantar and dorsiflexion along with summary results from the statistical tests are plotted in Figure 13. Significant site-dependent differences were seen throughout swing, at those phases were significant effects were also detected for ankle inversion/eversion (phases 9–11; Figure 13). During swing, a common feature for DF/PF was that FF Med differed from most other conditions. HL stimulation produced consistent reductions in dorsiflexion throughout swing and plantar and dorsiflexion during swing (phases 9–11).

## Discussion

In this paper we examined the site and phase-dependency of gait adaptation in response to non-noxious cutaneous stimulation at five discrete sites on the plantar foot surface. We thus evaluated the topographic organisation and neuromechanical reflex effects from cutaneous afferents innervating the foot sole. Our results suggest that cutaneous inputs from discrete regions on the foot sole stabilize and enhance coupling between afferent feedback from the foot sole and neuromechanical function during locomotion. Stimulation evoked clear phase and location-specific reflexes in muscles acting at the ankle and the topographic distribution of responses produced changes in forces under the foot and ankle kinematics. As described for electrical stimulation of whole cutaneous nerves innervating the foot, mechanical changes in kinematics were mostly found during swing and kinetic changes in forces under the foot during stance [4,11].

### Neuromechanical expression depends upon the phase of locomotion

Functional interpretations of the integrated neuromechanical responses are found below, organized within 4 functional phases of walking in which distinct responses occurred [3]: stance transition, stance, swing transition, and swing (see Figure 3).

### Stance transition

The dominant site-specific features of stimulation applied at the swing to stance transition were effects on TA muscle activity and force detected at the heel FSR. At the transition to stance, HL stimulation led to unloading at the heel FSR. Functionally, non-noxious cutaneous input at the heel may have increased TA activity to facilitate eversion if needed to avoid scuffing or tripping, and initiate weight acceptance by the stance limb. As ground contact is expected from swing to stance, tactile input from heel stimulation was likely interpreted as enhanced ground contact initiation. These observations are supported by research on late swing tibial nerve stimulation (innervating the heel, midfoot medial, and forefoot medial foot sole), which evoked a form of placing reaction characterized by ankle plantarflexion [4].

Effects with stimulation of sites distal to the heel may be perceived as uneven terrain requiring increased loading of the heel to accommodate the stance limb and is consistent with previous research demonstrating that stance phase sural nerve stimulation during walking produced dorsiflexion and eversion to accommodate for what could be perceived as uneven terrain along the lateral foot margin and near the heel [11]. With regard to M-M and F-M effects, although previous literature reports plantarflexion following tibial stimulation, there has been evidence of reflex reversal in Sol and TA muscle activity at a plantar boundary existing approximately midfoot [14]. This boundary exists along the foot sole’s lateral margin, but is likely mirrored along the medial margin. Heel stimulation during seated and standing isometric contractions respectively produced excitatory responses in Sol and inhibitory responses in TA, while forefoot medial and lateral stimulation resulted in the opposite effect [14]. This provides support for TA facilitation following F-M and F-L stimulation, but also for M-M stimulation given the reflex reversal boundary near the midfoot. M-M and F-M effects may thus simply reflect distinct reflexes that arise from activating the specific medial plantar branches of the tibial nerve.

At heel contact, stimulation delivered to the F-L site produced a significant increase in pressure at the Medial FSR, when compared to the decrease of force output with M-M stimulation. These effects may be the result of balance restoration and ankle stabilization in response to the increased tactile input from opposing plantar margins. Nakajima et al., [14] provide supporting evidence for decreased medial loading following F-L stimulation as each stimulation along the lateral plantar border facilitated ankle eversion. This interpretation makes functional sense, given the widespread facilitation of PL at this phase.

### Stance

The dominant effects of stimulation applied during early stance were changes in PL, MG, and TA muscle activity, forces in the medial and lateral FSRs, and ankle IN/EV. With regards to heel stimulation, as it has been shown that heel and tibial nerve stimulation produce plantarflexion, the increase in lateral loading may simply reflect a general increase in forefoot loading in response to what is perceived as uneven terrain [4]. This may be particularly evident during early stance as the lateral FSR is in contact with the ground at this phase. Additionally, inversion was typically produced by stimulation of all sites except the most medial midfoot site. During heel contact and early stance, tactile stimulation at the heel likely results in corrective changes due to an imbalance of pressure at the heel, thus altering motor output to promote readying of the stance limb for balanced weight support through an increase in forefoot pressure application. This would be reflected in the facilitation of TA (dorsiflexor and invertor) muscle found with medial stimulation and contrasted with facilitation of PL (dorsiflexor and evertor) muscle with lateral stimulation.

PL and MG muscles showed strong effects during stance along with changes in the force recorded from the medial FSR. Both M-L and F-L stimulation produced increased loading of the Medial aspect of the foot sole, indicating a shift in weight to ensure even loading of the foot in response to uneven terrain and thus improved balance. These effects are supported by Nakajima et al., [13,14]. The larger force output following M-L stimulation when compared to that of F-L may be indicative of greater forefoot stability during late stance when compared to the midfoot, as the body’s centre of mass is directly over the forefoot region in this phase.

### Swing transition

At the swing transition (phase 7) and into early swing (phase 8) we found modest effects of site-dependence. Generally, stimulation at lateral and distal sites (e.g. towards the forefoot) tended to enhance PL muscle activity and produce mixed results in MG muscle. TA muscle did show significant site dependence suggesting ankle plantar/dorsiflexion and inversion/eversion are main variables of control at this part of the step cycle. This was mirrored by forces under the heel FSR (generally increased from similar sites), as well as the medial and lateral FSR (generally reduced with stimulation). The overall impression is that regional plantar foot stimulation has a less specific regulatory role at the swing transition. This may be consistent with prior suggestion of general ground contact signalling (e.g. from distal tibial nerve) based upon whole cutaneous nerve stimulation [3-5].

### Swing

During swing (phases 9–11), PL, MG, and TA muscles showed dominant site-specific effects that were associated with changes in ankle IN/EV and DF/PF. Forefoot stimulation generally resulted in facilitation of all 3 muscles that produced eversion from medial and proximal sites and inversion elsewhere. This functional effect on inversion and eversion was differentially specified along the width and length of the foot such that F-L was always different from M-L and F-M. A related differential was also seen in DF/PF at the ankle during swing where forefoot stimulation tended to produce a DF response and more proximal stimulation a compensation at the ankle towards PF. Interestingly, stimulation at HL consistently produced PF. Taken together these can be interpreted as finely tuned obstacle avoidance to guide the foot away from perturbations during swing phase as previously described for whole nerve stimulation [4,11].

### Discrete activation of the sole produces “sensory steering” of foot motion during walking

The present observations support and extend to a locomotor context the earlier work of Nakajima and colleagues [13,14]. This earlier work in a postural context suggested a distinct organization of reflexes from the foot sole. The functional organization of the neural and mechanical responses evoked by stimulation of the foot sole are shown here to produce a kind of guided tuning—we suggest a “sensory steering”—of foot motion that accommodates to the perturbations mimicked by the electrical activation. A cartoon illustration of the general impressions from the synthesis of our data is shown in Figure 11. This illustration is not meant to be interpreted literally and also does not highlight any of the details of phase-dependency found in the figures above, but rather graphically shows the overall effects of stimulation.

**Figure 14:**
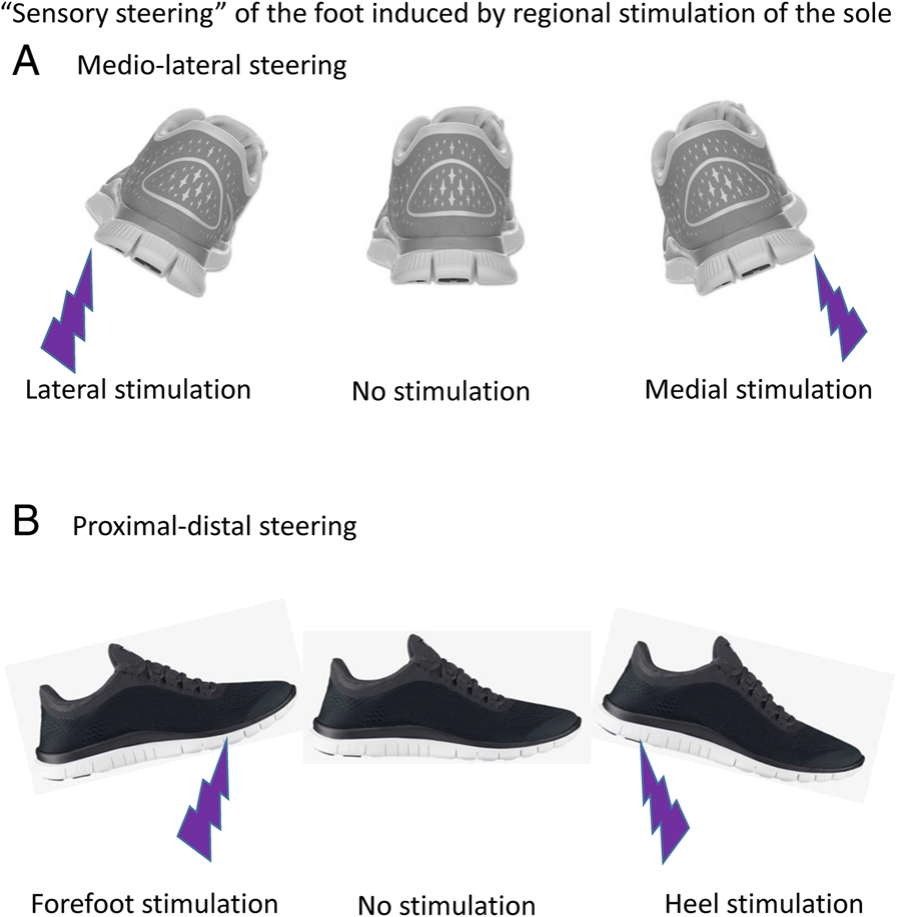
**Cartoon schematic illustrating the overall neuromechanical outcomes (“sensory steering”) found in the data.** Medio-lateral steering around a longitudinal axis is shown in **(A)** and proximal-distal steering along a transverse axis is shown in **(B)**. “Sensory steering” is as shown in this schematic is not meant as a literal representation of the amplitude of the evoked evokes at each phase of walking. Rather it is a general distillation of the overall neuromechanical outcomes found in our data.

The illustration in Figure 14 shows the general concept that activation under the foot sole produces a kind of tuned “sensory steering” response detected in EMG from the ankle muscles and manifesting itself mechanically by changes in ankle trajectory and pressure under the foot. The functional outcome of sensory steering is to guide foot trajectory away from or around the mimicked obstacle for foot placement. This overall interpretation is highly reminiscent of the earlier work of Arendt-Nielsen, Ole Andersen and colleagues who, in an elegant series of studies, showed a clear modular organization of nociceptive responses from the foot sole [22-26]. Our present results are consistent with a generally similar topographical and possibly modular organization of neuromechanical outcomes for both tactile and nociceptive sensory activation.In any case, with our tactile stimulation intensities, generally the neuromechanical outcomes could be seen as movement medio-lateral (with activation at the lateral and medial sites; Figure 14A), proximal-distal (with activation at the heel or forefoot; Figure 14B), and on the diagonal axis of the foot (comparing forefoot medial and midfoot lateral; not illustrated) depending upon the site of stimulation.

### Failure to observe differential effects of discrete stimulation in muscles distant from the ankle

In this study we observed distinct topographical effects of discrete foot sole stimulation only in the muscles acting predominately at the ankle (PL, MG, and TA). Muscles acting at the upper leg and hip (BF, VL, GM) and arm (PD) did not express this topography (see Figure 4). This was somewhat unexpected given the strong interlimb reflexes evoked in muscles across the body by cutaneous stimulation of the superficial peroneal [15,27] and sural [16] nerves. Interestingly, in static conditions Nakajima and colleagues also showed topographic effects in muscles acting at the ankle that were not observed in VL acting at the knee [14]. These observations could be a function of a difference in the quality of the perturbation represented by whole nerve versus discrete regional skin site stimulation. That is, discrete regional activation may represent smaller perturbations easily accommodated solely by responses controlling the ankle whereas whole nerve stimulation may be interpreted by the nervous system as a larger perturbation requiring more widespread responses across the body to avoid tripping and falling [3]. We await further research to clarify this issue.

### Methodological considerations with foot region stimulation

It must be pointed out that there is likely a major difference in constancy of stimulation input using the methodology of foot region stimulation during walking applied here and other studies using whole nerve stimulation. During stance the full body weight of each Participant put more pressure on stimulating electrodes on the foot than during swing, a difference likely to be absent with nerve stimulation. To help offset this concern, we created the low profile electrode interface and used 2 sided adhesive to keep the insole interface on the foot. We were most concerned with the insole “dropping” away from the foot during swing but the use of the adhesive was effective in this regard. In order to attempt a quantification of this, we determined the stimulator output needed to produce sensation at PT at all 5 stimulation sites in 3 conditions: standing (to mimic stance phase), sitting with feet on ground (to mimic partial body loading), and unloaded with the stimulated foot held off the ground (to mimic swing phase). Using standing as the “reference” position we found that the conditions with less body loading (sitting and mimicked swing phase) required stimulator outputs that ranged between 5 and 17% higher to achieve PT. Thus, this could lead to an small underestimation of the effects of stimulation from the M-M, M-L, F-M, and F-L regions compared to HL, or viewed conversely and overestimation of the relative effects of stimulation applied to HL. Despite that, we do not think this is a major concern with interpretation of our data since the actual evoked responses were an order of magnitude larger than those that would be anticipated by small changes in stimulation input. Indeed responses during swing (representing an unloaded condition) could often exceed those evoked during stance (loaded condition) (See Figure 4 for Peroneus Longus, Medial Gastrocnemius, and Tibialis Anterior).

## Conclusion

The results of this study further support suggestions that cutaneous nerves of the foot sole produce highly organised, topographic reflex effects in the lower limb of humans. Both site and phase dependence were observed in the kinetic responses, expressed as changes in force production at the foot sole, and offer additional evidence that non-noxious cutaneous perturbations applied to the bottom of the foot provide important tactile sensations for balance and maintenance of locomotion through the modulation of limb loading and foot placement [4,7,10]. While functional interpretation of cutaneous reflex effects on gait modulation have been determined for the dorsum of the foot [4,5,15] and with direct stimulation of tibial and sural nerve trunks [4,5], few studies have isolated cutaneous reflexes to specific regions of the foot sole [13,14]. The main findings of site and phase specificity do support previous literature and are important to providing further detail in our understanding of the topographical organisation of cutaneous reflexes.

This information is of importance to increase our understanding of how afferent feedback from specific cutaneous locations on the foot sole influences the mechanisms involved in locomotor output. This information may also have potential for rehabilitation strategies in impaired gait, such as in those arising after neurological damage. With a better understanding of how each receptive site on the plantar foot contributes to locomotion, researchers may be able to harness the effects of cutaneous reflexes to aid in enhancing functional modulation of gait following injury.

This study provides further evidence of site-specific and phase dependent gait modulation in response to non-noxious cutaneous stimulation at individual locations of the foot sole and could provide a better understanding of the behavioral relevancy, and potential rehabilitative use, of cutaneous input from specific regions on the plantar foot during locomotion.

## Competing interests

A portion of the funding for this project was obtained from a research contract-for-hire from NIKE Inc. One author (EPZ) has worked in the capacity as consultant for NIKE Inc. and one author (MN) is the current Director of the NIKE Sport Research Laboratory. We further certify and declare that none of these competing interests had any impact on the analysis, interpretation of results, or conclusions derived within the MS.

## Authors’ contributions

EPZ conceived the experiment, directed the analysis, and wrote the MS. TN, RAM, MN, and TK contributed to the experimental design, analysis, and interpretation. TB, TK and SM contributed to experimental design, conducted the experiments and participated in analysis of the data. All authors commented on and approved the final draft of the MS.

## Pre-publication history

The pre-publication history for this paper can be accessed here:

http://www.biomedcentral.com/2052-1847/6/33/prepub
